# Associations between partial pressure of oxygen and neurological outcome in out-of-hospital cardiac arrest patients: an explorative analysis of a randomized trial

**DOI:** 10.1186/s13054-019-2322-z

**Published:** 2019-01-28

**Authors:** Florian Ebner, Susann Ullén, Anders Åneman, Tobias Cronberg, Niklas Mattsson, Hans Friberg, Christian Hassager, Jesper Kjærgaard, Michael Kuiper, Paolo Pelosi, Johan Undén, Matt P. Wise, Jørn Wetterslev, Niklas Nielsen

**Affiliations:** 1 Department of Clinical Sciences Lund, Anesthesia and Intensive Care, Lund University, Helsingborg Hospital, S-251 87 Helsingborg, Sweden; 2 Clinical Studies Sweden, Skane University Hospital, Remissgatan 4, S-221 85 Lund, Sweden; 3 Department of Intensive Care, Liverpool Hospital, Locked Bag 7103, Liverpool BC, Sydney, NSW 1871 Australia; 4 Department of Clinical Sciences Lund, Neurology, Lund University, Skane University Hospital, Getingevägen 5, 221 85 Lund, Sweden; 5 Department of Clinical Sciences Lund, Anesthesia and Intensive Care, Lund University, Skane University Hospital, Getingevägen 5, 221 85 Lund, Sweden; 6 Department of Cardiology, Rigshospitalet, University of Copenhagen, DK 2100 Copenhagen, Denmark; 7 Department of Clinical Medicine, University of Copenhagen, DK 2100 Copenhagen, Denmark; 8 Intensive Care Unit, Leeuwarden Medical Centrum, Borniastraat 38, NL8934 AD Leeuwarden, Netherlands; 9 Department of Surgical Sciences and Integrated Diagnostics, University of Genoa, Genoa, Italy; 10 Department of Anesthesia and Intensive Care, IRCCS San Martino Policlinico Hospital, Genoa, Italy; 11 Department of Clinical Sciences Lund, Anesthesia and Intensive Care, Lund University, Hallands Hospital, S-30233 Halmstad, Sweden; 12 Adult Critical Care, University Hospital of Wales, Heath Park, Cardiff, CF144XW UK; 13 Copenhagen Trial Unit, Centre for Clinical Intervention Research, Dpt. 7812, Copenhagen University Hospital, Rigshospitalet, Blegdamsvej 9, DK-2100 Copenhagen, Denmark

**Keywords:** Out of hospital cardiac arrest, Partial pressure of oxygen, Cerebral performance, Biomarker, Serum tau

## Abstract

**Objective:**

Exposure to hyperoxemia and hypoxemia is common in out-of-hospital cardiac arrest (OHCA) patients following return of spontaneous circulation (ROSC), but its effects on neurological outcome are uncertain, and study results are inconsistent.

**Methods:**

Exploratory post hoc substudy of the Target Temperature Management (TTM) trial, including 939 patients after OHCA with return of spontaneous circulation (ROSC). The association between serial arterial partial pressures of oxygen (PaO_2_) during 37 h following ROSC and neurological outcome at 6 months, evaluated by Cerebral Performance Category (CPC), dichotomized to good (CPC 1–2) and poor (CPC 3–5), was investigated. In our analyses, we tested the association of hyperoxemia and hypoxemia, time-weighted mean PaO_2_, maximum PaO_2_ difference, and gradually increasing PaO_2_ levels (13.3–53.3 kPa) with poor neurological outcome. A subsequent analysis investigated the association between PaO_2_ and a biomarker of brain injury, peak serum Tau levels.

**Results:**

Eight hundred sixty-nine patients were eligible for analysis. Three hundred patients (35%) were exposed to hyperoxemia or hypoxemia at some time point after ROSC. Our analyses did not reveal a significant association between hyperoxemia, hypoxemia, time-weighted mean PaO_2_ exposure or maximum PaO_2_ difference and poor neurological outcome at 6-month follow-up after correction for co-variates (all analyses *p* = 0.146–0.847). We were not able to define a PaO_2_ level significantly associated with the onset of poor neurological outcome. Peak serum Tau levels at either 48 or 72 h after ROSC were not associated with PaO_2_.

**Conclusion:**

Hyperoxemia or hypoxemia exposure occurred in one third of the patients during the first 37 h of hospitalization and was not significantly associated with poor neurological outcome after 6 months or with the peak s-Tau levels at either 48 or 72 h after ROSC.

**Electronic supplementary material:**

The online version of this article (10.1186/s13054-019-2322-z) contains supplementary material, which is available to authorized users.

## Background

Survival after out-of-hospital cardiac arrest (OHCA) has improved over the last two decades and patients admitted to critical care are frequently discharged alive with increasingly good neurological outcome [1–4]. Following OHCA, patients regularly suffer from post cardiac arrest syndrome including symptoms of anoxic brain injury and reperfusion-related damage [5, 6]. In recent years, the optimal oxygen content in the post-cardiac arrest period has been a matter of debate since ventilation with high concentrations of oxygen after return of spontaneous circulation (ROSC) has been linked to worse outcome and an increased degree of cerebral neuronal damage in experimental cardiac arrest models [7–10]. In healthy volunteers, hyperoxemia decreases cerebral blood flow [11, 12], whilst hypoxemia is associated with the opposite effect [13]. Hyperoxemia also augments the production of reactive oxygen species (ROS), increases lipid oxidation and amplifies the inflammatory reaction in the brain during reperfusion after circulatory arrest [10, 14].

Clinical studies evaluating the impact of hyperoxemia and hypoxemia on neurological outcome after ROSC have shown inconsistent results when compared to the preclinical cardiac arrest models [15–19]. Two large landmark studies published in 2010 and 2011 limit their analysis to single or very few partial pressure of oxygen (PaO_2_) values in the post cardiac arrest phase [15, 17]. Although more recent studies have analysed multiple PaO_2_ values over time, so far, human studies continue to differ regarding patient selection, the use of targeted temperature management, outcome measurement, and methods of analysing blood gas and often lack a pre-defined sampling protocol [18–23].

We conducted this exploratory substudy of the prospectively collected blood-gas measurements in the Target Temperature Management after Out-of-Hospital Cardiac Arrest (TTM) trial in order to describe the fluctuation in PaO_2_ in the post-cardiac arrest phase and the association of hyperoxemia and hypoxemia with neurological outcome after 6 months [24]. An analysis of peak levels of serum Tau (s-Tau), a novel marker for neuronal injury, at either 48 or 72 h after ROSC and its association with PaO_2_ was subsequently performed to validate our results.

## Methods

The present study is a post hoc analysis of data acquired from 939 unconscious (Glasgow Coma Scale (GCS) < 8) adult (18 years or older) OHCA patients included in the TTM trial conducted between November 2010 and January 2013. Ethical committees in each participating country approved the TTM trial protocol, and informed consent was waived or obtained from all participants or relatives according to national legislations, in line with the Helsinki declaration.

The TTM trial was a randomized clinical trial recruiting patients in 36 intensive care units in Europe and Australia, designed to evaluate two target temperature regimes, 33 °C (*n* = 473) and 36 °C (*n* = 466), in unconscious adult OHCA patients after sustained ROSC [24]. Target temperature management was commenced at inclusion into the study. After 28 h, the patients were rewarmed to 37 °C core temperature over a period of 8 h and mandatory sedation was discontinued at the end of the 36-h intervention period. Resuscitation data was reported according to the Utstein style protocol [25]. Follow-up was obtained by structured face-to-face interview with the patient (86%) or structured telephone interview with the patient, care provider, or relative (14%) by a blinded assessor. The TTM trial did not show a significant difference between the two temperature groups in overall mortality at the end of the trial or in the composite of poor neurologic function or death at 180 days [24].

Baseline, intervention-related and physiological variables as demographic characteristics, comorbidities, pre-hospital and admission data, characteristics of the cardiac arrest, and baseline laboratory analyses were prospectively collected. A complete arterial blood gas analysis was performed in all patients at admission to hospital (T-1), start of intervention (T0), and after 4 (T4), 12 (T12), 20 (T20), 28 (T28), 32 (T32), and 36 (T36) hours post inclusion. In order to include the admission blood gas analysis, obtained after ROSC but before inclusion, we timed this analysis to 1 h before randomization in the statistical analysis of the present study. All arterial blood gases were collected according to an a priori designed protocol and analysed using the alpha-stat method only [26]. Median time from ROSC to inclusion was 133 (interquartile range 83–188) min. For the present study, PaO_2_ and FiO_2_ data were assessed and manually corrected for registration shortcomings by two authors in consensus (FE and NN). Details of the correction process are described in Additional file 1: Methods. Patient identification data were pseudomized. Patients who demised before the end of the intervention period were excluded from the present study to allow for a homogenous exposure period to PaO_2_. We chose to orientate on the STROBE Statement style for the study manuscript [27].

### Outcome

The primary outcome was overall neurological function at follow-up 6 months after cardiac arrest, assessed by Cerebral Performance Category (CPC) and dichotomized into good and poor with CPC 1 (good cerebral performance) and CPC 2 (mild neurological impairment) considered as good outcome, and CPC 3 to 5 as poor outcome with CPC 3–4 representing severe neurological impairment or vegetative state and CPC 5 death [28, 29]. In addition, we used the serum levels of Tau as a surrogate marker of neuronal injury in a subgroup analysis.

### Definition of hyperoxemia, hypoxemia, and normoxemia

We a priori defined hyperoxemia as a PaO_2_ > 40 kPa and hypoxemia as a PaO_2_ < 8 kPa in accordance with previous studies [15, 17, 23]. All values not defined as hyperoxemia or hypoxemia were defined as normoxemia.

### Primary analysis

#### Absolute oxygen levels

We divided our cohort according to the most extreme PaO_2_ of the individual patient into three groups: hyperoxemia, hypoxemia, and normoxemia. Thereafter, we compared the outcome of the hyperoxemia and hypoxemia group with normoxemia, followed by the comparison of each group’s outcome with the outcome of a composite group of the remaining patients.

#### Threshold analysis

In order to identify a possible PaO_2_ threshold value for the onset of the association of PaO_2_ and poor neurological outcome, we performed multivariable regression models with gradually increasing PaO_2_ levels.

### Secondary analyses

#### Oxygen exposure over time

To evaluate the cumulative PaO_2_ exposure over time, we formed a PaO_2_ over time integral from which we derived the time-weighted mean PaO_2_ (PaO_2_-TWM). Primarily, we evaluated the association of the PaO_2_-TWM from T-1 to T36 with outcome and, secondarily, from T-1 to T12 in order to identify effects of early hyperoxemia or hypoxemia.

#### Oxygen pressure difference

The difference between the most extreme PaO_2_ values during the observation time was calculated for each patient. The association of this maximum PaO_2_ difference with neurological outcome was analysed.

For an illustration of our primary and secondary analyses, see Additional file 1: Figure S1.

### Association between PaO_2_ and s-Tau

We used the cohort of 689 patients of the TTM trial substudy by Mattsson et al. [30] and evaluated the association of our multivariable PaO_2_ models with the highest level of s-Tau at either 48 or 72 h.

### Sensitivity analyses

Sensitivity analyses were performed with a complete case cohort of 468 patients with blood gas samples registered from all measuring points and an all-patient cohort of 922 patients including also those not surviving the full exposure period. Seventeen patients had insufficient data for analysis and were not included in the sensitivity analyses. Subsequently, we performed a sensitivity analysis of our primary analysis cohort including FiO_2_ as an additional co-variable and an all-cause mortality analysis.

## Statistics

Proportions are presented as percentages and continuous variables as mean with standard deviations (SD). Missingness was assumed at random [31]. Since the number of missing values exceeded 5%, we employed multiple imputation to compensate for the missing data [32]. Predictive mean matching, utilizing available non-missing values as well as available TTM trial study variables on the same individual and variables obtained from matching patients, was used. Twenty imputations were generated by chained equations and assessed by graphical methods. For each imputed dataset, PaO_2_ was evaluated using summary measures and regression models. The estimates from the regression for each imputed sample were combined into one estimate with 95% confidence intervals (CI) including the uncertainty from the multiple imputations based on Rubin’s rule [33]. Missing outcome data and death before end of intervention time entailed exclusion from analysis and was not compensated for.

Logistic regression analysis was used to assess the association between PaO_2_ and neurological outcome at 6-month follow-up. Results of our multivariable regression models are presented as odds ratios (OR) with 95% CI, OR describing continuous data present changes in one unit; for PaO_2_ 1 kPa, for pH one unit. All regression analyses were adjusted for pre-specified and in the context of OHCA relevant co-variates: age (years), sex (male/female), chronic heart failure (yes/no), asthma/chronic obstructive pulmonary disease (yes/no), cardiac arrest witnessed (yes/no), bystander cardiopulmonary resuscitation (yes/no), time to ROSC (minutes), Glasgow Coma Scale-Motor Score (1 vs 2–5), circulatory shock on admission (yes/no), first rhythm shockable (yes/no), and pH (units). We pooled the two temperature groups (33 °C and 36 °C) as there was no significant interaction between the PaO_2_ groups and the two temperature groups.

For the s-Tau analysis, multivariable linear regression was used and the depending variables were adjusted for the co-variables and interaction analyses as described above. After transforming the s-Tau values to a logarithmic scale, they were used as dependent variable in the linear regression analyses. The multiplicative change in s-Tau was depicted by the regression coefficients obtained for each independent variable after back transformation. Linear regression results are presented as beta-coefficient estimates with 95% CI.

The primary analyses were performed on a multiple imputation cohort as described above. The complete case and all-patient cohorts were used for sensitivity analysis. We regarded a two-sided *P* value < 0.05 as significant. Analyses were conducted using IBM SPSS statistics for Windows (version 22.0, Armonk NY) and R: A language and environment for statistical Computing (version 3.3.3 R Foundation for Statistical Computing, Vienna, Austria). The R package *mice* was used for multiple imputations [34].

## Results

**Fig. 1:**
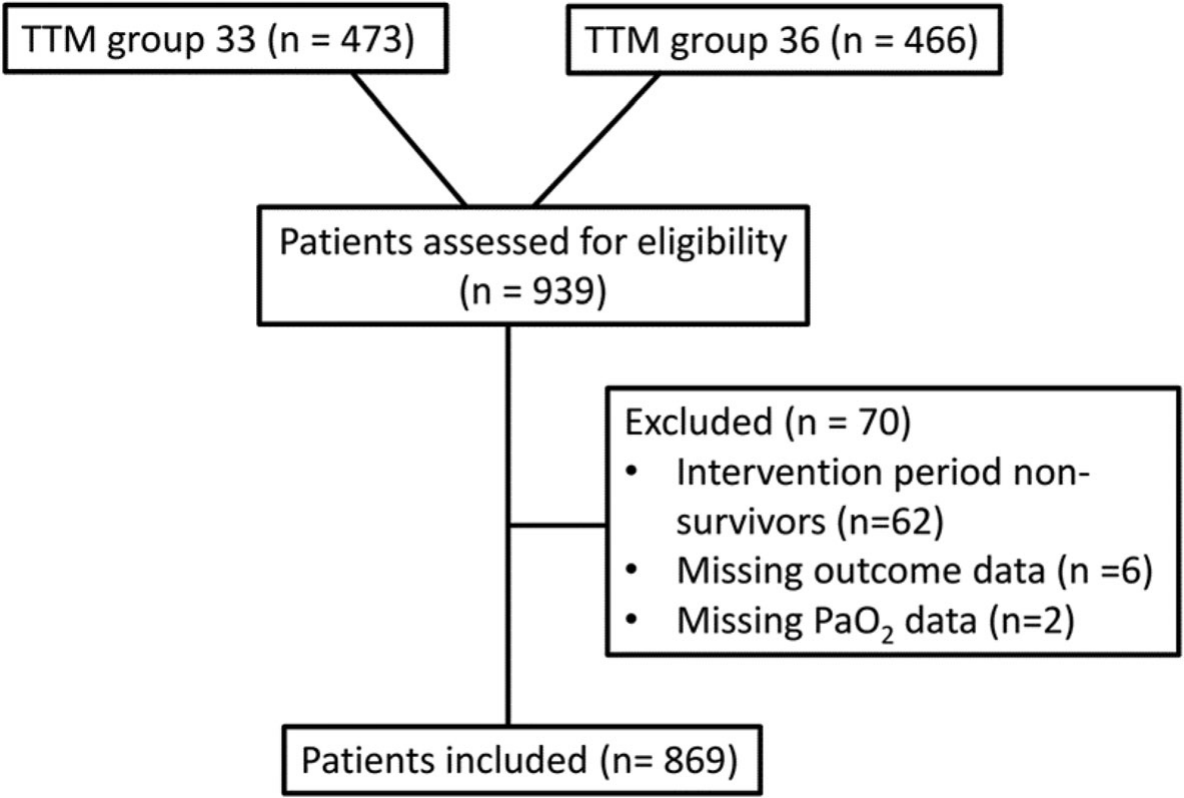
Patient selection pathway. TTM, targeted temperature management. TTM group, 33 or 36 °C core body temperature derived from the TTM trial [24]. *n*, number of patients. The diagram does not display the selection pathway for the s-Tau analysis

**Fig. 2:**
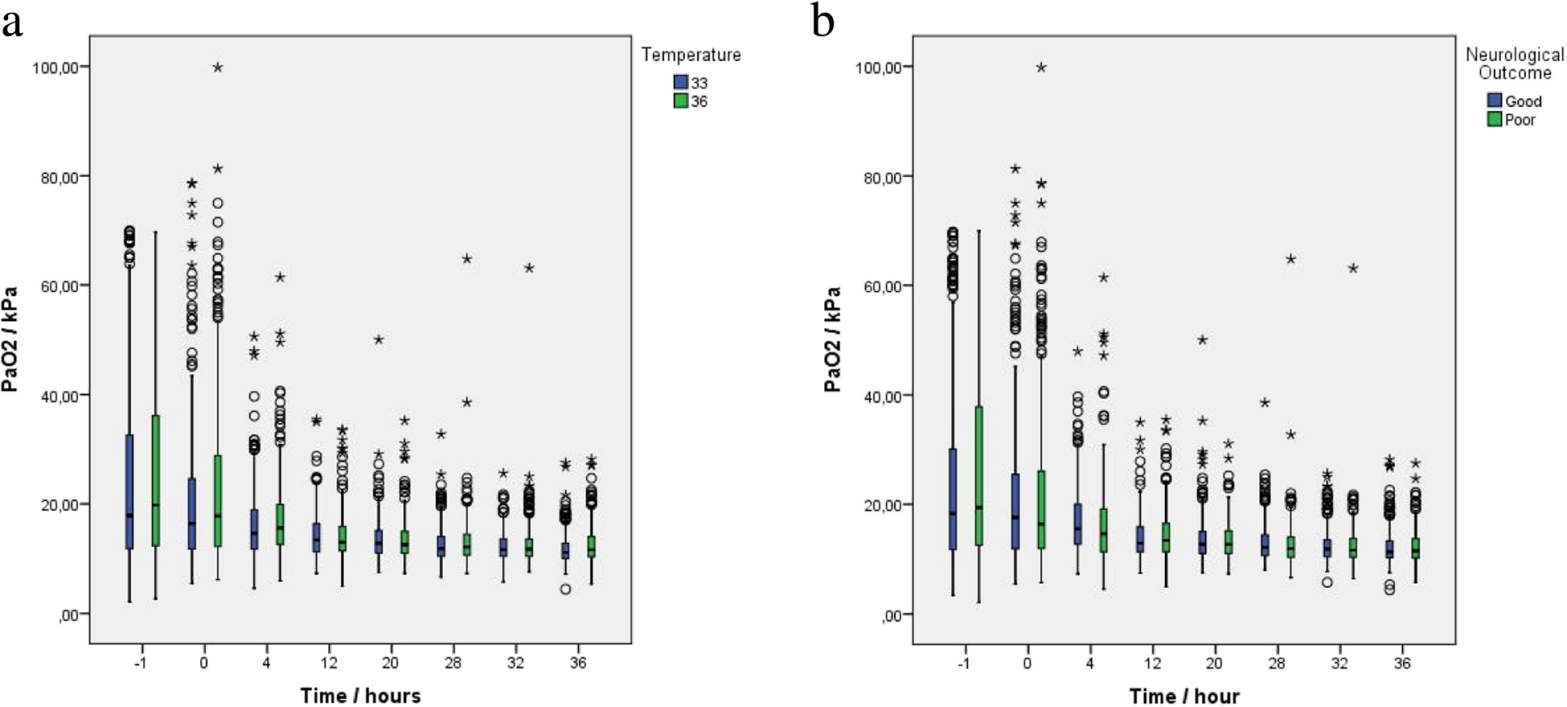
Boxplots depicting the distributional characteristics of PaO_2_ at 8 measurement points from admission to hospital to the end of intervention time for the TTM 33 and TTM 36 groups (**a**) and the investigated combined cohort dichotomized into good and poor outcome (**b**). Boxplot values are displayed as median, 25% quartiles from median and range. TTM, target temperature management. PaO_2_, partial arterial oxygen pressure. kPa, kilopascal. Core body temperature, 33 °C or 36 °C. White circle denotes the outliers. Asterisk denotes extreme outliers

Data for this explorative substudy was derived from 939 patients randomized in the TTM trial. We excluded patients who did not survive the intervention period (*n* = 62) and patients with no PaO_2_ data (*n* = 2) and missing neurological outcome data at 6-month follow-up (*n* = 6), which left 869 patients (92.5%) eligible for analysis (Fig. 1). Baseline characteristics for all the patients included and the different exposure groups are presented in Table 1. Overall, 441 patients (50.7%) had a good outcome whereas 428 patients (49.3%) had a poor outcome (Table 2). Of 869 patients, 384 (44.2%) died. Nine hundred eighteen of 6952 (13.2%) PaO_2_-measuring points were missing (Additional file 1: Table S1). At hospital admission, mean PaO_2_ was 25.1 (SD 17.0) kPa and diminished gradually in the temperature and outcome groups over time (Fig. 2a and b). In our primary analysis, we found that 199 of 869 (22.9%) patients were exposed to hyperoxemia at some point after admission to hospital, 112 (12.9%) were exposed to hypoxemia, 11 (1.3%) experienced both hyper- and hypoxemia, and 569 (65.5%) remained normoxic throughout. One hundred ninety-seven of 199 exposures to hyperoxemia occurred within the first 5 h after admission to hospital. Detailed post ROSC PaO_2_ data of the primary analysis groups are displayed in Table 3. In our secondary analyses, we found that PaO_2_-TWM from T-1 to T12 was mean 17.2 (SD 5.5) kPa whilst PaO_2_-TWM for all measurements was mean 14.5 (SD 3.2) kPa. The median maximum PaO_2_ difference was 14.3 kPa, with an interquartile range (IQR) of 8.6 to 27.2 kPa. For this study, we pooled the patients from the two TTM trial temperature groups into one cohort, which was feasible since the term of interaction analysis between the PaO_2_ exposure groups and TTM group affiliation (33 °C or 36 °C) showed no significant results (*p*_interaction_ = 0.537–0.972) (Additional file 1: Table S2).

**Table 1:** Baseline characteristics for all patients and exposure groups

Demographic characteristics	All patients *n* = 869	Hyperoxemia *n* = 199	Normoxemia *n* = 569	Hypoxemia *n* = 112	Hyper- and Hypoxemia *n* = 11
Age (years) (mean, SD)	63.9 ± 12.2	64.0 ± 12.8	63.9 ± 12.1	63.6 ± 11.9	63.7 ± 15.0
Male sex no. (%)	707 (81.4)	150 (75.4)	476 (83.7)	87 (77.7)	6 (58.4)
Background no. (%)					
Chronic heart failure	55 (6.3)	16 (8.0)	33 (5.9)	7 (6.2)	1 (11.0)
TIA or stroke	69 (8.0)	16 (7.8)	43 (7.5)	11 (9.7)	0 (0)
Arterial hypertension	347 (40.1)	78 (39.6)	228 (40.1)	46 (41.4)	5 (48.3)
Asthma/COPD	86 (9.9)	19 (9.7)	56 (9.9)	11 (9.8)	1 (5.3)
Diabetes mellitus	128 (14.8)	31 (15.8)	81 (14.3)	18 (16.3)	2 (22.0)
Previous PCI	101 (11.6)	23 (11.8)	61 (10.8)	18 (15.8)	1 (10.0)
Previous CABG	82 (9.5)	23 (11.5)	52 (9.2)	7 (6.5)	0 (0)
Cardiac arrest characteristics					
Bystander witnessed arrest no. (%)	783 (90.1)	174 (87.6)	514 (90.4)	104 (92.8)	9 (87.6)
Bystander CPR no. (%)	638 (73.4)	140 (70.3)	428 (75.1)	77 (69.1)	6 (61.7)
Circulatory shock on admission no. (%)	111 (12.8)	25 (12.4)	69 (12.1)	21 (18.9)	4 (36.4)
Prehospital intubation no. (%)	576 (67.2)	138 (69.7)	382 (68.2)	63 (57.2)	7 (62.7)
Time to ROSC (min) (mean, SD)	30.4 ± 21.7	30.9 ± 23.0	30.0 ± 21.5	31.6 ± 19.9	34.3 ± 15.5
Characteristics on admission					
pH (mean, SD.)	7.21 ± 0.15	7.20 ± 0.2	7.20 ± 0.1	7.10 ± 0.2	7.10 ± 0.2
PaCO_2_ (kPa) (mean, SD)	6.4 ± 2.0	6.1 ± 2.0	6.4 ± 1.9	7.3 ± 2.4	7.9 ± 2.6
PaO_2_ (kPa) (mean, SD)	25.1 ± 17.0	49.8 ± 15.1	18.9 ± 8.3	13.7 ± 11.1	35.0 ± 19.0
Lactate (mmol/L) (mean, SD)	6.5 ± 4.3	7.3 ± 4.1	6.0 ± 4.3	7.9 ± 4.5	10.2 ± 5.4
BE − 5 or less (mmol/l) no. (%)	579 (71.3)	144 (78.7)	362 (67.4)	81 (78.9)	7 (75.0)
GCS-Motor 1 no. (%)	443 (51.3)	116 (58.7)	280 (49.5)	55 (49.9)	7 (78.7)
Sedated on arrival no. (%)	254 (29.4)	43 (21.6)	179 (31.6)	34 (31.3)	2 (16.5)

% are displayed as valid percent over 20 imputations. Patients with combined exposure are also included in the separate hyperoxemia or hypoxemia exposure groups

*SD* standard deviation, *TIA* transient ischemic attack, *COPD* chronic obstructive pulmonary disease, *PCI* percutaneous coronary intervention, *CABG* coronary artery bypass graft, *CPR* cardiopulmonary resuscitation, *GCS* Glasgow Coma Scale, *ROSC* return of spontaneous circulation, *PaCO*_*2*_ arterial partial pressure of carbon dioxide, *PaO*_*2*_ arterial partial pressure of oxygen, *kPa* kilopascal, *mmol/l* millimoles per liter, *BE* base excess

**Table 2:** Neurological outcome according to CPC in the PaO_2_ exposure groups at 6-month follow-up

Exposure group	Good outcome	Poor outcome	Total
Hypoxemia	53 (47%)	59 (53%)	112
Hyperoxemia	88 (44%)	111 (56%)	199
Hypoxemia and hyperoxemia	2 (16%)	9 (84%)	11
Normoxemia	302 (53%)	267 (47%)	569

Patients with combined exposure are also included in the separate hyperoxemia or hypoxemia exposure groups

*CPC* cerebral performance category, *CPC 1–2* good outcome, *CPC 3–5* poor outcome

**Table 3:** Data on oxygen tension analyses in the post cardiac arrest period

Variable	All patients *n* = 869	Hyperoxemia *n* = 199	Normoxemia *n* = 569	Hypoxemia *n* = 112	Hyper- and hypoxemia *n* = 11
TWM-PaO_2_	14.0 (12.2–16.2)	15.9 (14.1–18.2)	13.8 (12.2–15.6)	12.2 (10.7–14.0)	13.90 (12.7–15.3)
TWM-PaCO_2_	5.3 (4.9–5.7)	5.3 (4.9–5.7)	5.3 (4.9–5.7)	5.4 (5.0–6.0)	5.4 (5.2–5.6)
PaO_2_ T-1	19.0 (12.1–33.8)	52.7 (42.7–61.4)	16.9 (12.1–24.5)	9.6 (7.3–15.5)	37.8 (20.3–49.8)
PaO_2_ T0	17.1 (11.8–25.7)	38.8 (21.2–52.9)	16.0 (11.8–22.0)	11.1 (8.4–16.5)	41.5 (22.0–52.2)
PaO_2_ T4	15.2 (12.2–19.7)	16.5 (13.1–21.3)	15.1 (12.4–19.6)	12.4 (9.8–17.1)	14.6 (11.1–17.8)
PaO_2_ T12	13.2 (11.3–16.2)	13.7 (11.5–16.6)	13.2 (11.3–16.3)	12.0 (10.3–14.9)	11.7 (9.6–14.9)
PaO_2_ T20	12.7 (11.0–15.1)	12.9 (11.3–15.5)	12.7 (11.0–15.0)	12.1 (10.2–14.5)	12.1 (10.0–15.0)
PaO_2_ T28	11.9 (10.5–14.2)	12.0 (10.4–13.9)	12.2 (10.7–14.4)	10.9 (9.7–13.5)	10.9 (9.8–12.8)
PaO_2_ T32	11.7 (10.4–13.7)	11.7 (10.3–13.7)	11.9 (10.6–13.8)	10.8 (9.4–12.3)	9.8 (8.2–11.4)
PaO_2_ T36	11.3 (10.2–13.5)	11.4 (10.2–13.5)	11.5 (10.3–13.7)	10.4 (8.9–11.8)	9.2 (8.3–10.6)
TWM pH	7.35 (7.31–7.39)	7.35 (7.32–7.39)	7.35 (7.31–7.39)	7.33 (7.28–7.37)	7.35 (7.31–7.39)
TWM PAW	12.5 (10.3–16.6)	12.6 (10.3–15.6)	12.6 (10.2–16.9)	11.9 (10.5–15.4)	12.9 (11.1–14.2)
TWM- BE	− 3.5 (−5.7 to − 1.5)	− 3.6 (− 5.9 to − 1.6)	− 3.6 (− 5.8 to − 1.5)	− 3.2 (− 5.2 to − 1.5)	− 3.65 (− 5.3 to − 2.2)
TWM-FiO_2_%	41.4 (34.9–49.7)	39.2 (33.8–45.0)	41.4 (34.7–49.4)	47.9 (39.0–59.3)	40.1 (34.3–50.6)
TWM-PaO_2_/FiO_2_	35.1 (26.8–43.8)	42.2 (34.2–50.0)	34.1 (26.8–42.3)	26.8 (20.4–34.7)	34.4 (27.2–43.3)

*PaO*_*2*_ arterial partial pressure of oxygen, *PaCO*_*2*_ arterial partial pressure of carbon dioxide. PaO_2_ and PaCO_2_ are displayed in kilopascal (kPa). *Hyperoxemia* PaO_2_ > 40 kPa, *Hypoxemia* PaO_2_ < 8 kPa, *Normoxemia* PaO_2_ values not defined hyper- or hypoxemia. *TWM* time weighted mean, *PAW* airway pressure, *BE* base excess, *FiO*_*2*_ fraction of inspired oxygen. Values are median with interquartile ranges (IQR). Patients exposed to hyper- and hypoxemia are also included in the separate hyperoxemia and hypoxemia exposure groups. *T* measuring time point in hours after inclusion into the TTM trial, *T-1* first blood gas analysis after admission but before inclusion

### Primary outcome analyses

**Fig. 3:**
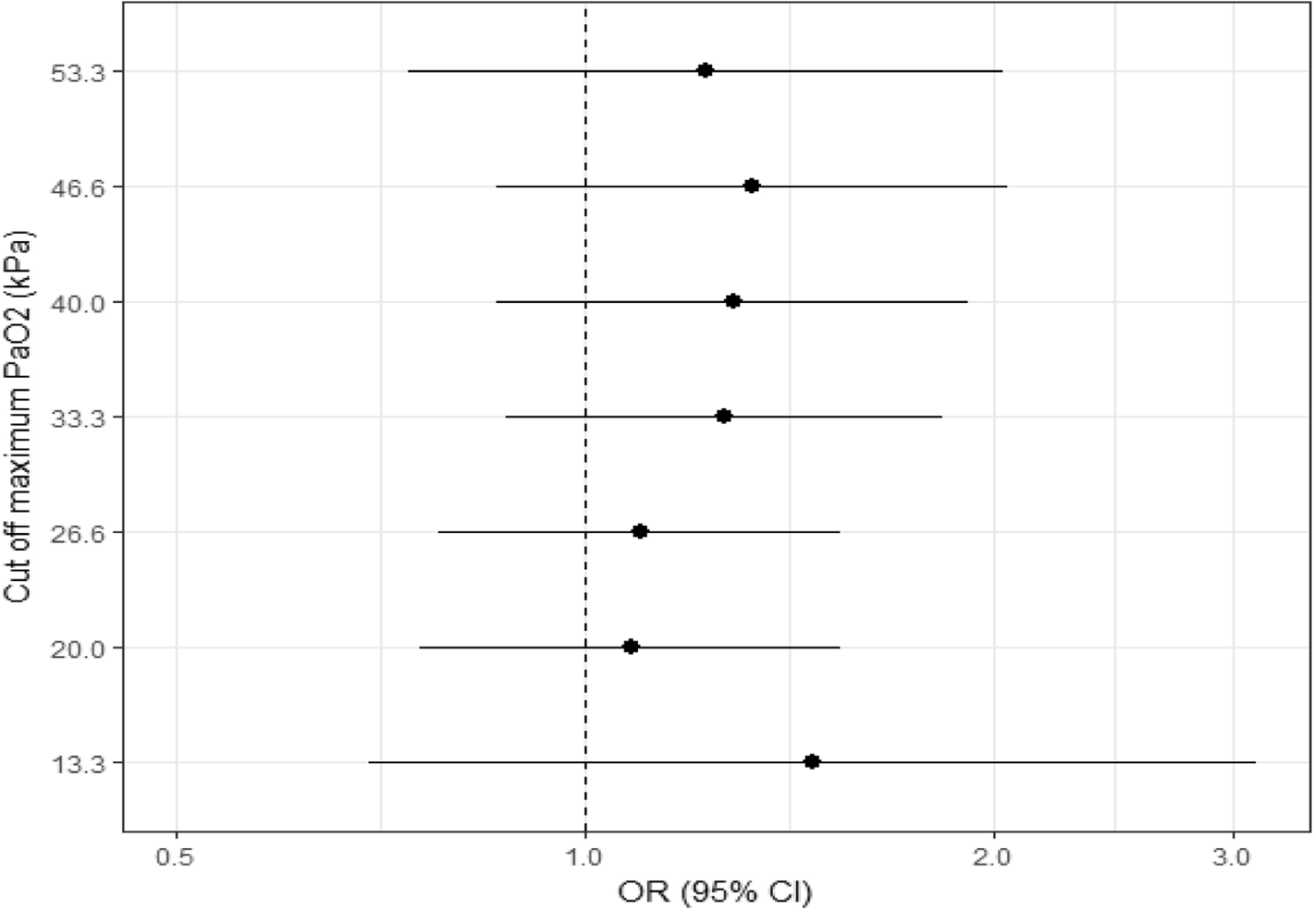
Forrest plot showing the adjusted OR’s (bullet points) with 95% CI’s (horizontal lines) for poor neurological outcome according to Cerebral Performance Category (CPC) for different PaO_2_ threshold values. OR, odds ratio. CI, confidence interval. PaO_2,_ partial pressure of oxygen. kPa, kilopascal. CPC, cerebral performance category. CPC 1-2, good outcome, CPC 3-5, poor outcome. ORs and CIs are presented on a logarithmic scale. OR above 1.0 indicates worse outcome above the PaO_2_ threshold and OR below 1.0 indicates better outcome above the PaO_2_ threshold

The absolute oxygen pressure analysis did not show a significant association between hyperoxemia versus normoxemia OR 1.24 (0.81, 1.89) *p* = 0.314 or hyperoxemia versus no hyperoxemia OR 1.28 (0.86, 1.91) *p* = 0.219 and poor neurological outcome. We also found no association with poor outcome in the hypoxemia exposure groups: hypoxemia versus normoxemia OR 1.06 (0.60, 1.85) *p* = 0.847 and hypoxemia versus no hypoxemia OR 1.13 (0.66, 1.91) *p* = 0.647. Detailed multivariate models of the hyperoxemia and hypoxemia analyses are presented in Tables 4 and 5. Figure 3 shows the adjusted ORs for poor neurological outcome of the PaO_2_ threshold analysis. We were not able to identify a PaO_2_ threshold value significantly associated with the onset of poor neurological outcome across gradually increasing PaO_2_ levels.

**Table 4:** Multivariate model of hyperoxemia versus normoxemia in relation to neurological outcome (CPC)

	OR	95% CI	*p* value
Hyperoxemia (normoxemia reference)	1.24	0.81–1.89	0.314
TTM group (33 °C reference)	0.99	0.70–1.41	0.976
Age (per year)	1.07	1.05–1.09	< 0.001
Sex (male reference)	1.36	0.85–2.17	0.200
Chronic heart failure (yes/no)	2.14	1.01–4.54	0.048
Asthma/COPD (yes/no)	1.29	0.70–2.36	0.410
Bystander witnessed arrest (yes/no)	0.61	0.35–1.07	0.087
Bystander CPR (yes/no)	0.88	0.58–1.34	0.550
Time to ROSC (per min)	1.03	1.02–1.04	< 0.001
GCS-Motor (1 vs 2–5)	0.40	0.28–0.57	< 0.001
Circulatory shock on admission (yes/no)	1.58	0.89–2.80	0.118
First rhythm shockable (yes/no)	0.19	0.11–0.34	< 0.001
pH (per unit increase)	0.38	0.10–1.49	0.164

*CPC* cerebral performance category, *CPC 1–2* good outcome, *CPC 3–5* poor outcome, *CI* confidence interval. *OR* odds ratio, *TTM* target temperature management, *COPD* chronic obstructive pulmonary disease, *CPR* cardiopulmonary resuscitation. *GCS-M* Glasgow Coma Scale-Motor, *ROSC* return of spontaneous circulation. OR < 1 indicates better outcome

**Table 5:** Multivariate model of hypoxemia versus normoxemia in relation to neurological outcome (CPC)

	OR	95% CI	*p* value
Hypoxemia (normoxemia reference)	1.06	0.60–1.85	0.847
TTM group (33 °C reference)	1.00	0.69–1.46	0.981
Age (per year)	1.06	1.04–1.08	< 0.001
Sex (male reference)	1.57	0.94–2.62	0.082
Chronic heart failure (yes/no)	1.94	0.87–4.34	0.106
Asthma/COPD (yes/no)	1.41	0.75–2.67	0.287
Bystander witnessed arrest (yes/no)	0.55	0.29–1.05	0.068
Bystander CPR (yes/no)	0.98	0.62–1.54	0.926
Time to ROSC (per min)	1.03	1.02–1.05	< 0.001
GCS—Motor (1 vs 2–5)	0.52	0.35–0.76	< 0.001
Circulatory shock on admission (yes/no)	2.41	1.34–4.34	0.003
First rhythm shockable (yes/no)	0.16	0.09–0.29	< 0.001
pH (per unit increase)	0.22	0.05–0.90	0.035

*CPC* cerebral performance category, *CPC 1–2* good outcome, *CPC 3–5* poor outcome, *CI* confidence interval, *OR* odds ratio, *TTM* target temperature management, *COPD* chronic obstructive pulmonary disease, *CPR* cardiopulmonary resuscitation, *GCS*-*M* Glasgow Coma Scale-Motor, *ROSC* return of spontaneous circulation. OR < 1 indicates better outcome

### Secondary outcome analyses

In our PaO_2_-TWM analyses, we did not find an association with poor neurological outcome, either for the complete exposure period (T-1 to T36), OR 1.03 (0.97, 1.09) *p* = 0.375, or for the early exposure period (T-1 to T12), OR 1.02 (0.98, 1.05) *p* = 0.288. We were also not able to show an association between maximum PaO_2_ difference and poor neurological outcome OR 1.01 (0.99, 1.02) *p* = 0.146.

### Association between PaO_2_ and s-Tau

Of the 689 patients in the s-Tau analysis, 64 were excluded as per our eligibility criteria and 36 had missing peak s-Tau levels at 48 or 72 h after ROSC, leaving 589 patients for analysis. Table 6 displays the detailed multivariable models of PaO_2_ and s-Tau. We did not find statistically significant associations between PaO_2_ and highest s-Tau at either 48 or 72 h after ROSC (*p* = 0.198–0.687).

**Table 6:** Peak s-Tau nested cohort analysis for the employed multivariable PaO_2_ models

Multivariable model	Estimate	95% CI	*p* value
Hypoxemia vs no-hypoxemia*	0.74	0.42–1.30	0.296
Hypoxemia vs normoxemia*	0.69	0.39–1.22	0.198
Hyperoxemia vs no-hyperoxemia*	1.19	0.78–1.82	0.419
Hyperoxemia vs normoxemia*	1.09	0.71–1.69	0.687
Maximum PaO_2_ difference**	1.01	0.99–1.02	0.436
PaO_2_-TWM T-1 to T36**	1.04	0.98–1.10	0.231
PaO_2_-TWM T-1 to T12**	1.02	0.98–1.05	0.391

*PaO*_*2*_ arterial partial pressure of oxygen, *CI* confidence interval, *s-Tau* serum Tau, *TWM* time-weighted mean, *T* measuring time point in hours after inclusion into the TTM trial, *T-1* first blood gas analysis after admission but before inclusion. For analyses of categorical data*, the estimate indicates how many times higher the s-Tau is compared to reference group. For analyses of continuous data**, the estimate indicates how much higher s-Tau is per 1 kPa PaO_2_ increase

### Sensitivity analyses

The analysis of the complete case cohort (*n* = 468) revealed non-significant results, in line with our multiple imputation cohort used for our primary analyses (*p* = 0.057–0.811). The all-patient cohort (*n* = 922), including also the patients dying during the exposure period, showed non-significant results (*p* = 0.060–0.979). The results with all-cause mortality instead of neurological function as the outcome were non-significant and similar to our primary analyses (*p* = 0.307–0.969). Adding FiO_2_ as a confounder to our primary analyses cohort did not significantly alter outcome (*p* = 0.102–0.793). Details of the sensitivity analyses are displayed in Additional file 1: Tables S3–S6. FiO_2_ and PaO_2_ were weakly correlated (*r* = − 0.23).

## Discussion

In this exploratory post hoc substudy of the TTM trial, we investigated the association of PaO_2_ outside normal ranges in the post cardiac arrest phase with poor neurological outcome 6 months after OHCA and peak s-Tau at either 48 or 72 h after ROSC. We found that 35% of patients were exposed to hyperoxemia or hypoxemia following ROSC. We did not find statistically significant associations between exposure to hyperoxemia or hypoxemia with poor neurological outcome at 6-month follow-up. The PaO_2_-TWM and maximum PaO_2_ difference analyses did not show an association with neurological outcome. Our findings did not indicate a PaO_2_ threshold value associated with the onset of poor neurological outcome. We were not able to detect an association between PaO_2_ and peak s-Tau at either 48 or 72 h after ROSC.

Our data shows that exposure to PaO_2_ outside the normal range, especially hyperoxemia, was common and most pronounced in the first hours after admission. This probably reflects clinical practice to continue ventilation with high FiO_2_ after ROSC, thus causing a propensity for early hyperoxemia, despite recommendations to titrate FiO_2_ to a target SpO_2_ of 94–98% to avoid hyperoxemia [35].

A recent observational multicentre study by Roberts et al. [23] found that exposure to early hyperoxemia, defined by two protocol-directed blood gas samples within the first 6 h after ROSC, was independently associated with poor neurological outcome at hospital discharge, corroborating results from a previous retrospective study by the same group [15]. Additionally, they identified PaO_2_ ≥ 40kPa as the threshold value for the association between poor neurological outcome and PaO_2_. Our primary hyperoxemia analyses are comparable since hyperoxemia exposure occurred almost exclusively early and the cut-off values of our threshold analysis are akin, but we did not confirm the findings by Roberts et al. In contrast to our study, their cohort was smaller, including in- and out-of-hospital cardiac arrest patients, follow-up was shorter, and exposure to hyperoxemia was more common (38% versus 23%).

Oxygen exposure over the first 24 h in a cohort of 409 OHCA patients treated with hypothermia to 33 °C was investigated in a prospective observational study by Vaahersalo et al. [22], showing in agreement with our time-weighted mean analyses, no association between oxygen exposure over time and neurological outcome. However, several aspects of this study make direct comparison with our study difficult; in the study by Vaahersalo et al., only 6% of patients were exposed to a PaO_2_ > 40 kPa, and blood gases were not obtained by protocol and analysed by pH and alpha-stat methods instead of alpha-stat only.

The physiological cerebral vascular response to hyperoxemia is vasoconstriction, alteration of cerebral blood flow (CBF), and subsequently reduced regional oxygen delivery [36, 37]. A study by Voicu et al. indicates that this mechanism might be impaired in a proportion of OHCA patients [38]. Two blood gas management strategies, alpha-stat versus pH-stat, were investigated and revealed an absence of change in CBF velocities between the two modalities in non-survivors compared to survivors. Furthermore, the non-survivor group also showed no difference in jugular vein oxygen saturation, arteriojugular oxygen content, and cerebral oxygen extraction. This study identifies a subgroup of OHCA patients with failed cerebral vascular autoregulation and increased risk for secondary brain injury due to possible cerebral hyperperfusion and unregulated hyperoxemia exposure. Similar subgroups have been described in previous studies [39, 40], highlighting the heterogeneity of OHCA cohorts.

In a hypoxic state, neurons are unable to utilize oxidative phosphorylation and are forced to resort to glycolysis for ATP production which is a short lived rescue mechanism before the onset of neuronal cell injury and death [41]. This primary, hypoxic injury occurs during OHCA and is together with the secondary, reperfusion injury that begins immediately after ROSC regarded as one of the major contributors to the post cardiac arrest syndrome [6, 13], whilst the effects of prolonged hypoxemia after ROSC are undetermined. In a non-OHCA cohort, well-trained acclimatized climbers were fully functional at PaO_2_ levels as low as 2.54 kPa [42], and in a canine model EEG readings were normal at PaO_2_ values of 2.6 kPa, provided that no CBF impairment was present [43]. Mechanisms of physiological acclimatization to hypoxia and unimpaired CBF are implausible in adult OHCA patients due to increased age and co-morbidities. Hence, the PaO_2_ threshold for the onset of hypoxic neuronal demise is presumably above the presented extreme values, but also likely to be significantly lower than the 8 kPa cut off employed in our and previous investigations, which provides an explanation for the deviating results of studies investigating hypoxemia [15, 17, 20, 21].

In the present study, we did not find an association between PaO_2_ and peak s-Tau levels, which supports the lack of association between PaO_2_ and neurological outcome. S-Tau is in the context of OHCA, a novel biomarker for neuronal injury, and the 48- and 72-h peak level is a significantly better clinical predictor for neurological outcome after 6 months than neuron-specific enolase (NSE) or clinical information alone [30].

In summary, clinical observational studies investigating hyperoxemia and hypoxemia differ in outcome, possibly due to patient selection, sampling and analysis methodology, and random error. Observational studies are not showing associations with a better outcome after hyperoxemia or hypoxemia exposure, but clinical randomized trials randomizing patients to PaO_2_ values outside normal ranges are lacking and would be a plausible next step to further investigate the influence of PaO_2_ on outcome.

## Study limitations and strengths

In this study, we employed different analytic approaches to test the association of serial PaO_2_ measurements in resuscitated comatose patients after OHCA with functional parameters and biomarkers as outcome. The present study was conceived after completion of the TTM trial, and due to the nature of this exploratory, post hoc substudy, all results must be regarded as hypothesis generating and we cannot make causality statements from our findings. Considering the direction of the ORs and the widths of the CIs of our analyses, we cannot rule out possible associations. Threshold values for the detrimental effects of hyperoxemia or hypoxemia are undetermined; therefore, we accepted values in keeping with previous studies. For this study, we hypothesized that oxygen pressure in-between PaO_2_ measurement was linear and we were not able to account for short-term variations of PaO_2_. FiO_2_ management in the primary study was not protocolized and at the physician’s discretion. Our study nevertheless has considerable strengths. The investigated cohort of 939 OHCA was homogenous and large, and patients were selected from a multicenter randomized clinical trial with liberal inclusion criteria and a trial protocol reflecting standard practice. All physiological and biochemical parameters were collected prospectively, according to a pre-defined time-based protocol, eliminating measurement bias. Blood gases were analysed by a uniform method. Our results were adjusted for in the context of OHCA important confounders. The findings of this study were strengthened by an all-patient, a complete case, and an all-cause mortality sensitivity analysis, additionally supported by using a biomarker. The association of PaO_2_ outside normal ranges after OHCA with a biomarker of neurological injury has to our knowledge not previously been investigated.

Follow-up data was acquired using a structured protocol, with a majority performed face-to-face, and a minimal loss of patients in the follow-up period [24].

## Conclusion

Although exposure to hyperoxemia and hypoxemia following OHCA was common in this study, we found hyperoxemia, hypoxemia, time-weighted mean oxygen exposure, and maximum partial pressure of oxygen difference not to be independently associated with neurological outcome at 6-month follow-up or with s-Tau at either 48 or 72 h after ROSC. Our findings did not indicate a PaO_2_ threshold value for the onset of poor neurological outcome.

## Additional file

Additional file 1: Associations between partial pressure of oxygen and neurological outcome in out-of-hospital cardiac arrest patients: an explorative analysis of a randomized trial. Additional details on study methods, explanatory figure and tables depicting detailed information on missing patients, interaction analysis, and sensitivity analyses. (DOCX 82 kb)

## Abbreviations

AUC: Area under the curve
BE: Base excess
CABG: Coronary artery bypass graft
CBF: Cerebral blood flow
COPD: Chronic obstructive pulmonary disease
CPC: Cerebral performance category
CPR: Cardio pulmonary resuscitation
FiO_2_: Fraction of inspired oxygen
GCS: Glasgow Coma Scale
ICU: Intensive care unit
kPa: Kilopascal
NSE: Neuron-specific enolase
OHCA: Out-of-hospital cardiac arrest
OR: Odds ratio
PaO_2_: Partial pressure of oxygen
PCI: Percutaneous coronary intervention
ROSC: Return of spontaneous circulation
SD: Standard deviation
S-Tau: Serum Tau
TIA: Transient ischemic attack
TTM: Target temperature management
TWM: Time-weighted mean
