# Supplementary material for: Associations between partial pressure of oxygen and neurological outcome in out-of-hospital cardiac arrest patients: an explorative analysis of a randomized trial
Source: Crit Care. 2019 Jan 28;23:30. doi: 10.1186/s13054-019-2322-z (PMC6348606; doi:10.1186/s13054-019-2322-z)
Supplement: Supplementary file 1 — Associations between partial pressure of oxygen and neurological outcome in out-of-hospital cardiac arrest patients: an explorative analysis of a randomized trial. Additional details on study methods, explanatory figure and tables depicting detailed information on missing patients, interaction analysis, and sensitivity analyses. (DOCX 82 kb) [file 13054_2019_2322_MOESM1_ESM.docx]

**Associations between partial pressure of oxygen and neurological outcome in out-of-hospital cardiac arrest patients: an explorative analysis of a randomized trial**

ADDITIONAL FILE 1

Florian Ebner, Susann Ullén, Anders Åneman, Tobias Cronberg, Hans Friberg, Christian Hassager, Jesper Kjærgaard, Michael Kuiper, Niklas Mattsson, Paolo Pelosi, Johan Undén, Matt P Wise, Jørn Wetterslev and Niklas Nielsen

For submission to *Critical Care*

**Methods**

**Data correction**

The total number of measuring points was 7512. 174 (2.3%) measuring points were manually corrected. Corrections were performed strictly in context with other measurements before and after the erroneous measurement and other physiological parameters registered for the same individual. The majority of corrections, 166 (95%) concerned the incorrect registration of oxygen as a fraction instead of in percent (e.g. 0.4 instead of 40%). In 7 of the of the corrected cases, a PaO_2_ value was registered with the decimal placed in the wrong position (e.g. 88.7 kPa instead of 8.87). In one case FiO_2_ and PaO_2_ were interchanged.

**Figure S1.**

Explanatory illustration depicting the most extreme PaO_2_ value as maximum or minimum PaO_2_ (primary analysis) as well as the maximum PaO_2_ difference as the distance between the maximum and minimum PaO_2_ values (secondary analysis). The grey area under the chart represents the area under the curve from which the time weighted mean PaO_2_ (PaO_2_-TWM) was calculated: individual PaO_2_ pressure level over time in-between measuring points (kPa x h) / total time from admission (T-1) to end of intervention (T36) (h) = PaO_2_-TWM (kPa) (secondary analysis). The PaO_2_ threshold analysis is not illustrated in Figure S1. *PaO_2_ = partial pressure of oxygen. kPa = kilopascal. T -1 = first blood gas analysis after admission but before inclusion into the TTM-trial. T0 to 36 = time points (hours) for protocolized blood gas sampling after inclusion into the TTM-trial.*

**Results**

| **Table S1. Number of missing measurements at each time point. Total no. n = 869** | | | | | | | | |
| --- | --- | --- | --- | --- | --- | --- | --- | --- |
| Time (h) | T -1 | 0 | 4 | 12 | 20 | 28 | 32 | 36 |
| Missing n | 57 | 160 | 95 | 93 | 122 | 123 | 141 | 127 |
| % of total | 6.6 | 18.4 | 10.9 | 10.7 | 14.0 | 14.2 | 16.2 | 14.6 |

*n = number. h = hours. T -1 = time at admission, after ROSC but before randomization*

| **Table S2. Term of interaction analysis between primary PaO_2_ analysis groups divided into 33°C and 36°C temperature groups and neurological outcome (CPC).** | | | |
| --- | --- | --- | --- |
|  | **OR** | **95% CI** | **p-value** |
| Hyperoxemia vs normoxemia | 0.84 | 0.36 - 1.96 | 0.689 |
| Hyperoxemia vs no hyperoxemia | 0.95 | 0.41 - 2.13 | 0.905 |
| Hypoxemia vs normoxemia | 0.92 | 0.30 - 2.74 | 0.878 |
| Hypoxemia vs no hypoxemia | 1.07 | 0.39 - 2.90 | 0.902 |
| PaO_2_-TWM T-1 to T12 | 1.00 | 0.92 - 1.08 | 0.912 |
| PaO_2_-TWM T-1 to T36 | 1.05 | 0.90 - 1.22 | 0.537 |
| Maximum PaO_2_ difference | 1.00 | 0.97 - 1.03 | 0.972 |

*OR = odds Ratio. CI = confidence Interval. PaO_2_ = partial arterial oxygen pressure. TWM = time weighted mean. T = measuring time point in hours after inclusion into the TTM-trial. T -1 = first blood gas analysis after admission but before inclusion. CPC = cerebral performance category, CPC 1-2 =good outcome, CPC 3-5 = poor outcome. OR < 1 indicates better outcome. vs = versus.*

| **Table S3. Sensitivity analysis. Neurological outcome according to CPC in the complete cases PaO_2_ exposure groups (n=468) at 6 months follow-up adjusted for confounders.** | | | |
| --- | --- | --- | --- |
| Analysis | **OR** | **95% CI** | **p-value** |
| Hyperoxemia vs normoxemia | 1.31 | 0.74 – 2.29 | 0.343 |
| Hyperoxemia vs no hyperoxemia | 1.47 | 0.85 – 2.52 | 0.167 |
| Hypoxemia vs normoxemia | 0.73 | 0.32 - 1.65 | 0.450 |
| Hypoxemia vs no hypoxemia | 0.91 | 0.43 - 1.92 | 0.811 |
| PaO_2_-TWM T-1 to T12 | 1.03 | 0.98 – 1.09 | 0.148 |
| PaO_2_-TWM T-1 to T 36 | 1.04 | 0.96 – 1.13 | 0.316 |
| Maximum PaO_2_ difference | 1.01 | 0.99 – 1.03 | 0.057 |

*n = number. OR = odds Ratio. CI = confidence Interval. PaO_2_ = partial arterial pressure of oxygen. TWM = time weighted mean. T = measuring time point in hours after inclusion into the TTM-trial. T -1 = first blood gas analysis after admission but before inclusion. CPC = cerebral performance category, CPC 1-2 =good outcome, CPC 3-5 = poor outcome. OR < 1 indicates better outcome. vs = versus. We adjusted for the following confounders: age (years), sex (male/female), chronic heart failure (yes/no), asthma/chronic obstructive pulmonary disease (yes/no), cardiac arrest witnessed (yes/no), bystander CPR (yes/no), first rhythm shockable (yes/no), time to ROSC (minutes), GCS-Motor Score (1 versus 2 - 5), shock on admission (yes/no), pH at admission (units).*

| **Table S4. Sensitivity analysis. Neurological outcome according to CPC in the all-patients PaO_2_ exposure groups at 6-month follow-up adjusted for confounders.** | | | |
| --- | --- | --- | --- |
| Analysis | **OR** | **95% CI** | **p-value** |
| Hyperoxemia vs normoxemia | 1.12 | 0.73 – 1.70 | 0.604 |
| Hyperoxemia vs no hyperoxemia | 1.18 | 0.79 - 1.77 | 0.415 |
| Hypoxemia vs normoxemia | 0.93 | 0.52 – 1.63 | 0.791 |
| Hypoxemia vs no hypoxemia | 1.01 | 0.59 – 1.71 | 0.979 |
| PaO_2_-TWM T-1 to T12 | 1.01 | 0.98 – 1.05 | 0.434 |
| PaO_2_-TWM T-1 to T36 | 1.04 | 0.99 – 1.09 | 0.060 |
| Maximum PaO_2_ difference | 1.01 | 0.99 – 1.02 | 0.146 |

*n = number. OR = odds Ratio. CI = confidence Interval. PaO_2_ = partial arterial pressure of oxygen. TWM = time weighted mean. T = measuring time point in hours after inclusion into the TTM-trial. T -1 = first blood gas analysis after admission but before inclusion. CPC = cerebral performance category, CPC 1-2 =good outcome, CPC 3-5 = poor outcome. OR < 1 indicates better outcome. vs = versus. Patients with no outcome data (n=6), no PaO_2_ data (n=7) and lack of confounders (n=4) were excluded, 922 patients were included in the final analysis. We adjusted for the following confounders: age (years), sex (male/female), chronic heart failure (yes/no), asthma/chronic obstructive pulmonary disease (yes/no), cardiac arrest witnessed (yes/no), bystander CPR (yes/no), first rhythm shockable (yes/no), time to ROSC (minutes), GCS-Motor Score (1 versus 2 - 5), shock on admission (yes/no), pH at admission (units).*

| **Table S5. Sensitivity analysis. Neurological outcome according to CPC in the primary analysis PaO_2_ exposure groups at 6-month follow-up (n = 869), adjusted for confounders and FiO_2_.** | | | |
| --- | --- | --- | --- |
| Analysis | **OR** | **95% CI** | **p-value** |
| Hyperoxemia vs normoxemia | 1.28 | 0.83 – 1.98 | 0.261 |
| Hyperoxemia vs no hyperoxemia | 1.34 | 0.88 – 2.03 | 0.160 |
| Hypoxemia vs normoxemia | 1.08 | 0.61 – 1.91 | 0.793 |
| Hypoxemia vs no hypoxemia | 1.11 | 0.65 – 1.88 | 0.693 |
| PaO_2_-TWM T-1 to T12 | 1.02 | 0.98 – 1.06 | 0.182 |
| PaO_2_-TWM T-1 to T36 | 1.03 | 0.97 – 1.10 | 0.246 |
| Maximum PaO_2_ difference | 1.01 | 0.99 – 1.02 | 0.102 |

*n = number. OR = odds Ratio. CI = confidence Interval. PaO_2_ = partial arterial pressure of oxygen. TWM = time weighted mean. CPC = cerebral performance category, CPC 1-2 =good outcome, CPC 3-5 = poor outcome. OR < 1 indicates better outcome. vs = versus. We adjusted for the following confounders: age (years), sex (male/female), chronic heart failure (yes/no), asthma/chronic obstructive pulmonary disease (yes/no), cardiac arrest witnessed (yes/no), bystander CPR (yes/no), first rhythm shockable (yes/no), time to ROSC (minutes), GCS-Motor Score (1 versus 2 - 5), shock on admission (yes/no), pH at admission (units). FiO_2_ = fraction of inspired oxygen.*

| **Table S6. Sensitivity analysis. All-cause mortality analysis in the primary analysis PaO_2_ exposure groups (n=869), adjusted for confounders.** | | | |
| --- | --- | --- | --- |
| Analysis | **OR** | **95% CI** | **p-value** |
| Hyperoxemia vs normoxemia | 1.17 | 0.76 – 1.79 | 0.481 |
| Hyperoxemia vs no hyperoxemia | 1.24 | 0.82 – 1.87 | 0.307 |
| Hypoxemia vs normoxemia | 0.99 | 0.55 – 1.76 | 0.969 |
| Hypoxemia vs no hypoxemia | 1.10 | 0.65 – 1.88 | 0.725 |
| PaO_2_-TWM T-1 to T12 | 1.00 | 0.97 – 1.03 | 0.941 |
| PaO_2_-TWM T-1 to T36 | 0.99 | 0.94 – 1.04 | 0.690 |
| Maximum PaO_2_ difference | 1.00 | 0.99 – 1.02 | 0.443 |

*n = number. OR = odds Ratio. CI = confidence Interval. PaO_2_ = partial arterial pressure of oxygen. TWM = time weighted mean. CPC = cerebral performance category, CPC 1-2 =good outcome, CPC 3-5 = poor outcome. OR < 1 indicates better outcome. vs = versus. We adjusted for the following confounders: age (years), sex (male/female), chronic heart failure (yes/no), asthma/chronic obstructive pulmonary disease (yes/no), cardiac arrest witnessed (yes/no), bystander CPR (yes/no), first rhythm shockable (yes/no), time to ROSC (minutes), GCS-Motor Score (1 versus 2 - 5), shock on admission (yes/no), pH at admission (units).*
